# A case of recurrent acute acalculous cholecystitis in a patient with renal cell carcinoma treated with pazopanib and axitinib: case report and literature review

**DOI:** 10.3389/fphar.2026.1928976

**Published:** 2026-09-09

**Authors:** Xiaolu Zeng, Jing Zhang, Chen Chen, Xueru Sun, Lei Yang

**Affiliations:** Department of Cancer Center, The First Hospital of Jilin University, Changchun, Jilin, China

**Keywords:** acute acalculous cholecystitis, anti-angiogenic tyrosine kinase inhibitor, axitinib, pazopanib, renal cell carcinoma, TKI

## Abstract

A 74-year-old male patient with renal cell carcinoma developed disease progression in the left adrenal gland and lungs 12.8 years after surgery. He presented with symptoms of cholecystitis 4 weeks after starting pazopanib and 8 weeks after starting axitinib. The symptoms were relieved after treatment with drug discontinuation, anti-infective therapy and symptomatic supportive care. The Naranjo Adverse Drug Reaction Probability Scale scores showed 4 points for pazopanib and 6 points for axitinib, indicaing a probable association of the event with the above two drugs. Meanwhile, a literature review was conducted by retrieving studies on tyrosine kinase inhibitor-induced cholecystitis from the PubMed database from 2005 to 2025, followed by systematic collation and summary. The study suggests that anti-angiogenic tyrosine kinase inhibitors may induce rare episodes of cholecystitis in specific patient populations. Clinicians should maintain adequate vigilance, conduct close clinical monitoring, and adjust therapeutic regimens in a timely manner to ensure patient safety and optimize prognosis.

## 1 Introduction

Renal cell carcinoma (RCC) is a malignant tumor originating from renal tubular epithelium, accounting for approximately 90% of renal malignancies (Bex et al., 2025). Clear cell renal cell carcinoma (ccRCC) represents the most common histological subtype of renal cancer, comprising approximately 70%–75% of renal carcinoma histological subtypes (Bukavina et al., 2022). Surgical resection remains the primary treatment option for localized clear cell renal cell carcinoma, yet patients still face risks of recurrence and metastasis postoperatively (Chen et al., 2009). The advent of tyrosine kinase inhibitors (TKIs) has significantly improved the prognosis for patients with advanced clear cell renal cell carcinoma. Among these, pazopanib and axitinib, as representative multi-target TKIs, exert their antitumor effects by specifically inhibiting targets such as vascular endothelial growth factor receptors (VEGFR), thereby blocking tumor angiogenesis (Gotink and Verheul, 2010). They have been widely adopted in clinical first- or second-line treatments.

The adverse reactions of pazopanib and axitinib commonly include hypertension, hematologic toxicity, skin reactions, gastrointestinal reactions, and liver function impairment (Li et al., 2025). However, the adverse reaction of inducing cholecystitis is extremely rare for both drugs. Currently, there are few relevant case reports domestically and internationally, and its mechanism of occurrence remains incompletely understood. Typical clinical manifestations of cholecystitis include right upper quadrant pain, nausea, vomiting, and fever (Gallaher and Charles, 2022). Failure to promptly recognize and intervene may lead to severe consequences such as acute suppurative cholecystitis, gallbladder perforation, or even death, significantly impacting treatment progress and quality of life.

This report describes a case of ccRCC in which the patient developed cholecystitis symptoms following treatment with both pazopanib and axitinib. By reviewing relevant literature, we explore the clinical characteristics and management strategies for this rare adverse reaction. The aim is to enhance clinicians’ awareness of such uncommon adverse effects associated with these drugs and provide guidance for safe clinical use.

## 2 Case presentation

The patient, a 74-year-old male, was healthy with no prior history of hepatobiliary disease. A left renal mass was detected during a physical examination in 2011, leading to a partial left nephrectomy in September 2011. Postoperative pathology revealed clear cell renal cell carcinoma of the left kidney, with hemorrhage and cystic changes, measuring approximately 3.0 × 3.0 × 2.0 cm. Immunohistochemistry results included CgA (−), CD10 (+), Syn (−), P53 (−), EGFR (−), VEGF (−), Inhibin (−), K-ras (−), Ki-67 (<1%+). The final diagnosis was stageI (pT_1a_N_0_M_0_) ccRCC. Regular follow-up was conducted for the first 3 years postoperatively and ceased after 3 years, with no evidence of significant disease progression observed during the follow-up period.

A July 2024 follow-up renal computed tomography (CT) revealed an occupying lesion in the left adrenal region measuring approximately 6.6 × 4.3 cm. The patient underwent partial adrenalectomy at an external hospital. The postoperative pathology report indicated: clear cell malignant tumor of the left adrenal gland with necrosis. Immunohistochemistry results included Vimentin (+), Inhibin (−), CgA (−), Syn (−), Ki-67 (20%), CAIX (+), CD10 (+), PAX-8 (+), RCC (partially +), P504S (+), CK7 (−). No further antitumor therapy was administered postoperatively.

**FIGURE 1:**
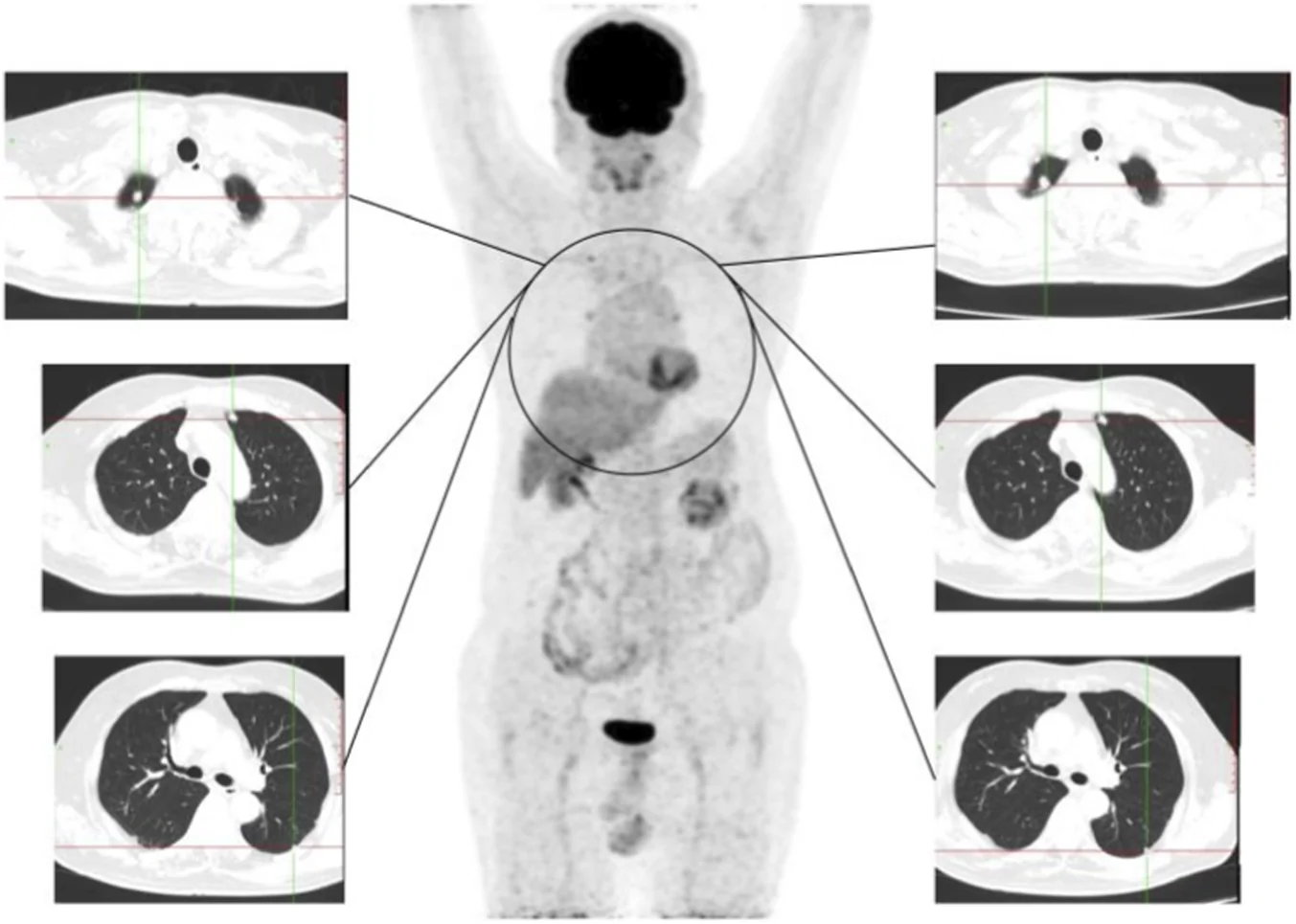
PET-CT Partial Scan Results: Multiple Pulmonary Nodules for the patient from August 2024 (left)vs. September 2025 (right).

**FIGURE 2:**
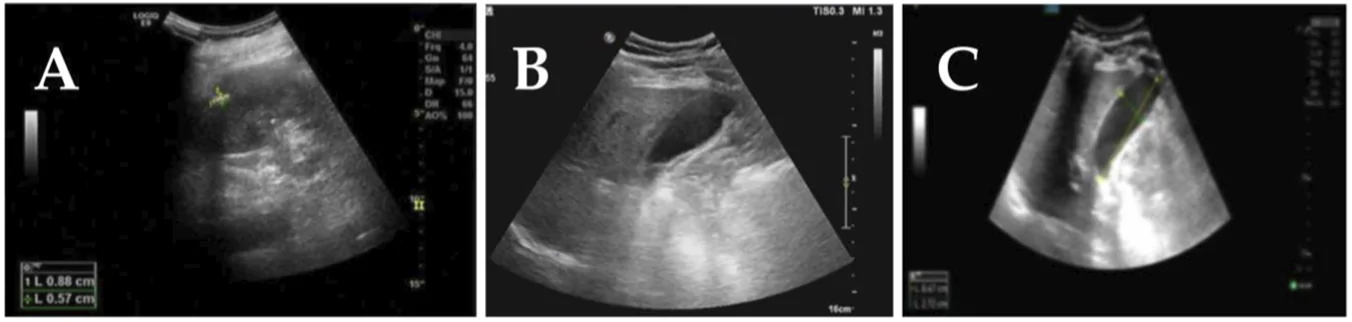
Abdominal Ultrasound results for the patient **(A)** September 2024: The gallbladder measured 90 mm × 39 mm with a wall thickness of 4 mm and irregular mural surface; fine punctate intraluminal deposits can be seen. **(B)** November 2025: The gallbladder is normal in size with mural thickening measuring 10 mm, demonstrating the double-wall sign. **(C)** July 2026: The gallbladder measured 85 mm × 27 mm with a mural thickness of 2 mm and a smooth wall.

One month postoperatively, positron emission tomography/computed tomography (PET/CT) revealed multiple nodules in both lungs, measuring approximately 0.3–0.7 cm (Figure 1), StageIV(pT_x_N_0_M_1_) ccRCC. Treatment initiated with pazopanib 800 mg once daily. During the third week, the patient developed mild thrombocytopenia. The pazopanib dose was adjusted to 600 mg once daily. During the fourth week, the patient suddenly developed pain in the right upper abdomen accompanied by fever, and tested positive for Murphy’s sign. Persistent elevations of AST (peak 291.8 U/L) and ALT (peak 142.9 U/L) were observed in this patient, accompanied by elevated bilirubin (maximal TBIL 42.4 μmol/L, DBIL 11.1 μmol/L); mild subsequent rises in GGT (134 U/L peak) and ALP (92.7 U/L peak), as well as moderate thrombocytopenia with a platelet nadir of 41 × 10^9^/L, were also documented. Baseline liver function tests and platelet counts prior to medication initiation were all within normal ranges: AST 30.6 U/L, ALT 22.4 U/L, GGT 30.0 U/L, ALP 82.4 U/L, TBIL 13.2 μmol/L, DBIL 2.8 μmol/L, PLT 238 × 10^9^/L. Abdominal ultrasound revealed cholecystitis and cholestasis (Figure 2A). Although baseline abdominal ultrasound was not performed prior to medication initiation, baseline abdominal CT demonstrated no chronic hepatobiliary abnormalities. Pazopanib was immediately discontinued, and the patient was hospitalized. Magnetic resonance cholangiopancreatography (MRCP) confirmed cholecystitis. MRCP demonstrated no significant dilatation of the intra- and extrahepatic bile ducts. The common bile duct measured approximately 0.7 cm in diameter, with mild luminal narrowing at its suprapancreatic segment and no definite intraluminal lesions. The gallbladder was unremarkable in size with mild wall thickening, and no abnormal intraluminal signal intensities were visualized. Symptoms improved following antimicrobial therapy, hepatoprotective treatment, and symptomatic management. Targeted therapy was not resumed thereafter.

A follow-up PET-CT performed in September 2025 showed that the nodules in both upper lung lobes had increased in size compared with previous scans, with the largest measuring approximately 1.3 × 1.0 cm (Figure 1). Subsequently, treatment with oral axitinib 5 mg twice daily was initiated. One month into treatment, the patient developed hoarseness and fatigue, along with elevated thyroid-stimulating hormone (TSH). TSH concentrations increased from 3.001 μIU/mL to 19.404 μIU/mL, whereas free triiodothyronine (FT3) and free thyroxine (FT4) stayed within normal reference ranges at all time points. The dose was adjusted to 3 mg twice daily. Symptoms improved after dose reduction, but gallbladder wall edema recurred 1 month later (Figure 2B), prompting immediate discontinuation of the drug. Follow-up ultrasound 2 weeks later showed resolution of gallbladder wall edema. Subsequently, TSH concentrations normalized to 2.499 μIU/mL.

**FIGURE 3:**
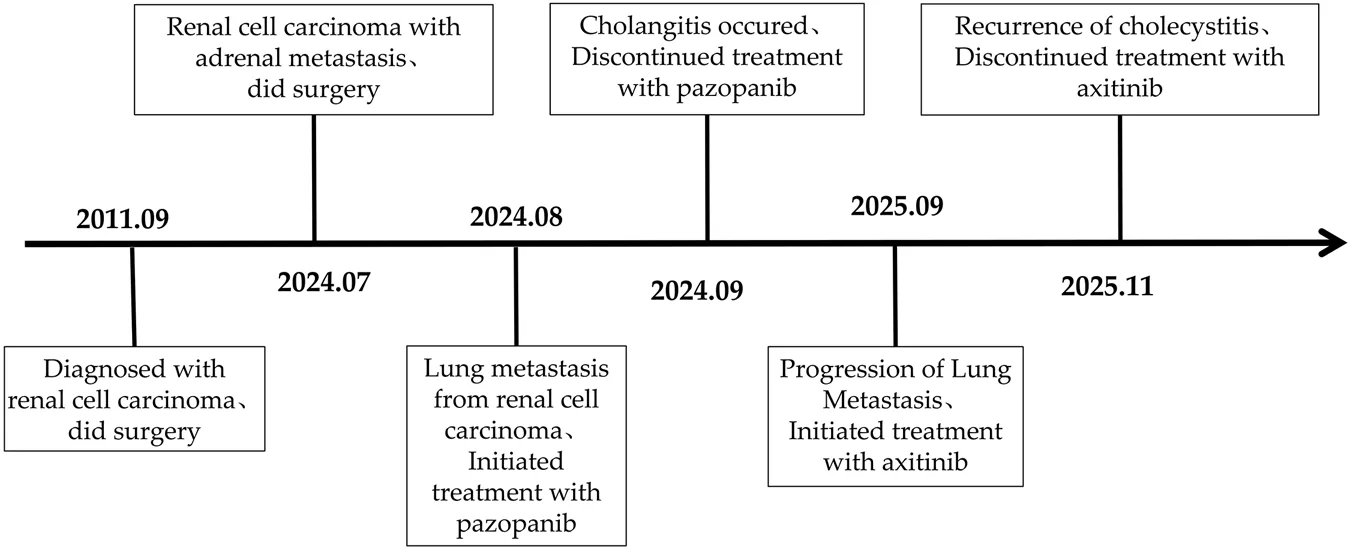
Clinical course of the patient in this case.

Furthermore, white blood cell (WBC) counts remained within the normal reference range across both cholecystitis episodes, peaking at 6.87 × 10^9^/L. Serial C-reactive protein (CRP) levels trended downward from 133.59 mg/L to 33.82 mg/L and procalcitonin (PCT) levels decreased from 0.59 ng/mL to 0.2 ng/mL during the initial episode, with two sets of blood cultures returning negative results. The second cholecystitis was detected on abdominal ultrasound, as the patient remained asymptomatic and no concurrent CRP or PCT testing was conducted. A surgical consultation was arranged during the first cholecystitis attack, and the surgical team concluded immediate operative intervention was unnecessary. At the latest follow-up, laboratory tests showed that all biochemical abnormalities noted during prior treatment had resolved, with all indices remaining steadily within normal reference ranges. Follow-up abdominal ultrasound (Figure 2C) revealed a gallbladder of normal dimensions with a smooth wall 2 mm thick. To date, the patient has elected to cease anticancer therapy and receives routine surveillance for pulmonary metastases. No recurrent cholecystitis has been documented since drug discontinuation. The clinical course and timeline of the patient are shown in Figure 3.

## 3 Discussion and literature review

The recurrence rate following partial nephrectomy for stageIRCC ranged from 3% to 5%, with most recurrences developing within 5 years postoperatively and a 10-year recurrence rate of 0.6% (Kim et al., 2024). This patient presented with stageI (pT_1a_N_0_M_0_) RCC that recurred 12.8 years after partial nephrectomy. Postoperative follow-up was conducted with every 6 months for the first 2 years, followed by annual visits thereafter. The patient underwent regular follow-up for the first 3 years with no evidence of disease progression; however, regular follow-up was discontinued after the third year. Accordingly, extended surveillance for ≥10 years should be implemented even among patients who undergo partial nephrectomy for early-stage RCC.

Common sites of metastasis for RCC include the lungs, bones, lymph nodes, liver, adrenal glands, and brain (Dudani et al., 2021). Therefore, comprehensive physical examinations during follow-up are essential. With the continuous advancements in imaging technology, follow-up should incorporate CT and magnetic resonance imaging (MRI) scans, with PET/CT utilized when necessary (Woon et al., 2024). During this patient’s follow-up period, only urinary system color Doppler ultrasound was performed, with no attention paid to pulmonary status. Both the timing and methods of follow-up were inadequate.

At recurrence, the patient developed adrenal and pulmonary metastases but had a favorable International Metastatic Renal Cell Carcinoma Database Consortium (IMDC) risk profile. Accordingly, following adrenalectomy, targeted therapy with pazopanib at 800 mg daily was initiated. However, cholecystitis-related symptoms emerged in the fourth week of treatment. At that time, we did not consider a causal relationship between the cholecystitis and pazopanib. Subsequently, the symptoms worsened, leading to drug discontinuation. After supportive symptomatic treatment, the cholecystitis symptoms resolved. Following further progression of the pulmonary metastases, treatment with axitinib (5 mg twice daily) was initiated. Unfortunately, cholecystitis recurred again in the eighth week of treatment. Both episodes of cholecystitis in this patient occurred as adverse reactions following administration of small-molecule TKIs, prompting our investigation. We used the Naranjo Adverse Drug Reaction Probability Scale (Tables 1, 2) to assess whether the acute cholecystitis was causally related to pazopanib and axitinib, as this scale is specifically designed to evaluate the likelihood of drug-related adverse events. The Naranjo Adverse Drug Reaction Probability Scale scores showed 4 points (Table 1) for pazopanib and 6 points (Table 1) for axitinib, indicaing a probable association of the event with the above two drugs. No cases of pazopanib-associated acute cholecystitis have been previously reported, whereas only one case related to axitinib has been documented. In 2017, Kameda T (Kameda et al., 2017)reported a 71-year-old male with lung metastases following partial nephrectomy for renal cell carcinoma. He developed abdominal pain (pneumoenteritis) on day 24 of axitinib treatment, leading to discontinuation. On day 16 after discontinuation, the patient developed fever and abdominal pain. MRCP revealed acute acalculous cholecystitis. Symptoms improved with conservative management; neither percutaneous transhepatic gallbladder drainage (PTGBD) nor cholecystectomy was performed.

**TABLE 1:** Adverse drug reaction probability scale (Naranjo scale) with pazopanib in the present case.

Title 1	Score
Are there previous conclusive reports on this reaction?	0
Did the adverse event appear after the suspected drug was given?	2
Did the adverse reaction improve when the drug was discontinued or a specific antagonist was given?	1
Did the adverse reaction appear when the drug was re-administered?	0
Are there alternative causes that could have caused the reaction?	0
Did the reaction reappear when a placebo was given?	0
Was the drug detected in any body fluid in toxic concentrations?	0
Was the reaction more severe when the dose was increased, or less severe when the dose was decreased?	0
Did the patient have a similar reaction to the same or similar drugs in any previous exposure?	0
Was the adverse event confirmed by any objective evidence?	1
Scoring	4

**TABLE 2:** Adverse drug reaction probability scale (Naranjo scale) with axitinib in the present case.

Title 1	Score
Are there previous conclusive reports on this reaction?	1
Did the adverse event appear after the suspected drug was given?	2
Did the adverse reaction improve when the drug was discontinued or a specific antagonist was given?	1
Did the adverse reaction appear when the drug was re-administered?	0
Are there alternative causes that could have caused the reaction?	0
Did the reaction reappear when a placebo was given?	0
Was the drug detected in any body fluid in toxic concentrations?	0
Was the reaction more severe when the dose was increased, or less severe when the dose was decreased?	0
Did the patient have a similar reaction to the same or similar drugs in any previous exposure?	1
Was the adverse event confirmed by any objective evidence?	1
Scoring	6

A comparison between this case report and other cases of cholecystitis associated with multi-targeted TKIs is shown in Table 3. According to relevant literature reports on TKI-induced cholecystitis retrieved from the PubMed database between 2005 and 2025, cholecystitis first appeared in the fourth week after axitinib administration (Kameda et al., 2017), at an average of 3.8 weeks (range: 2–5 weeks) after sunitinib administration (Choi et al., 2020; Furubayashi et al., 2014; da Fonseca et al., 2014; Nakano et al., 2012; Gomez-Abuin et al., 2009; de Lima Lopes and Rocha Lima, 2007), in the fourth week after sorafenib administration (Aihara et al., 2012), and in the 12th week after lenvatinib administration (Honda et al., 2020). For RCC, both single-agent small-molecule TKIs and combination immunotherapy serve as foundational treatments, with the control of tumor progression being paramount. Given this therapeutic dilemma, could cholecystectomy mitigate drug-induced gallbladder injury, reduce cholecystitis-related complications, improve patient quality of life, and extend the duration of antitumor drug administration? In 2014, da Fonseca LG (da Fonseca et al., 2014) described a 55-year-old patient with stageIVRCC with lung metastases. After receiving sunitinib at a dose of 50 mg for 4 weeks on and 2 weeks off, the patient developed acute acalculous cholecystitis in the fourth week of treatment. Immediate drug discontinuation and anti-infective therapy were initiated promptly. Subsequently, cholecystectomy was performed due to severe sepsis. Treatment was resumed 3 weeks after surgery and maintained for 6 months until disease progression, with no recurrence of severe gallbladder-related adverse events during this period. Honda S (Honda et al., 2020) reported in 2020 a 59-year-old patient with stageIVliver cancer bone metastases who experienced gallbladder perforation after increasing lenvatinib dosage from 4 mg/day to 8 mg/day and the patient developed gallbladder perforation 3 months later. Conservative management, including drug discontinuation and anti-infective therapy, led to clinical improvement. After 2 months of lenvatinib withdrawal, the patient resumed lenvatinib treatment and gradually increased the dosage back to 8 mg/day. One month later, gallbladder perforation recurred, necessitating permanent discontinuation of lenvatinib. Conservative management with anti-infective therapy controlled the perforation; however, the patient died 48 days later due to disease progression. These cases suggest that when TKI-induced cholecystitis or gallbladder perforation occurs, clinicians may consider surgical intervention based on the patient’s specific condition. Additionally, it raises the question of whether TKIs therapy can be resumed after cholecystectomy to control tumor disease progression. When drug-induced cholecystitis occurs and TKIs discontinuation is not feasible, cholecystectomy may currently represent a suitable therapeutic strategy.

**TABLE 3:** Cases of cholecystitis induced by applied TKIs as reported.

No.	Sex/Age(y)	Primary tumor/stage	Application of TKIs^1^	The time when cholecystitis first appeared(w)	Treatment of cholecystitis	Reinitiation of TKIs
1. This patient	M/74	RCC^2^/stage IV	Pazopanib 800 mg/day	4	Discontinuation of the drugs therapy and antibiotics	No
			Axitinib 10 mg/day	8		
2. (Kameda et al., 2017)	M/71	RCC/stage IV	Axitinib	4	Antibiotics, bowel rest, and discontinuation of the axitinib therapy	Not mentioned
3. (Choi et al., 2020)	M/64	RCC/stage IV	Sunitinib 50 mg/day for 2 weeks on and 1 week off	5	Discontinuation of the Sunitinib therapy, PTGD^3^ and Cholecystectomy	Yes, 37.5 mg/day for 2 weeks on and 1 week off
4. (Furubayashi et al., 2014)	M/66	RCC/stage IV	Sunitinib 50 mg/day for 4 weeks on and 2 weeks off	2	Switch to Axitinib, PTGD and Cholecystectomy	Not mentioned
​	F/67	RCC/stage IV	Sunitinib 37.5 mg/day for 4 weeks on and 2 weeks off	2	Discontinuation of the Sunitinib therapy, antibiotics and Cholecystectomy	Not mentioned
5. (da Fonseca et al., 2014)	M/55	RCC/stage IV	Sunitinib 50 mg/day for 4 weeks on and 2 weeks off	4	Discontinuation of the Sunitinib therapy, antibiotics and Cholecystectomy	Yes, 50 mg/day for 4 weeks on and 2 weeks off
6. (Nakano et al., 2012)	F/75	RCC	Sunitinib 50 mg/day for 4 weeks on and 2 weeks off	4	Discontinuation of the Sunitinib therapy, PTGD and Cholecystectomy	No
7. (Gomez-Abuin et al., 2009)	F/62	RCC	Sunitinib 50 mg/day for 4 weeks on and 2 weeks off	4	Discontinuation of the Sunitinib therapy and antibiotics	Yes, 37.5 mg/day for 4 weeks on and 2 weeks off Ultimately discontinued due to tumor progression
8. (de Lima Lopes and Rocha Lima, 2007)	M/50	GIST^4^	Sunitinib 50 mg/day for 4 weeks on and 2 weeks off	4	Antibiotics and percutaneous cholecystostomy	Yes, Sunitinib 50 mg/day for 4 weeks on and 2 weeks off
9. (Aihara et al., 2012)	F/43	HCC^5^	Sorafenib 400 mg/day	4	Discontinuation of the Sorafenib therapy and prednisolone (500 mg/d) for 3 days	Not mentioned
10. (Honda et al., 2020)	F/59	HCC/stage IV	Lenvimab 4 mg/day gradually increase to 8 mg/day	12	Discontinuation of the drugs therapy and antibiotics	Yes, but cholecystitis recurred, Lenvimab was permanently discontinued

1 TKIs: tyrosine kinase inhibitors.

2 RCC: renal cell carcinoma.

3 PTGBD: percutaneous transhepatic gallbladder drainage.

4 GIST: Gastrointestinal Stromal Tumor

5 HCC: hepatocellular carcinoma.

The mechanism by which anti-angiogenic TKIs induce acute acalculous cholecystitis remains incompletely understood, but it is speculated that this may be related to the inhibitory effect of TKIs on bile duct cell proliferation, their anti-angiogenic activity, and their promotion of microthrombus formation (Choi et al., 2020). Drug accumulation stimulates gallbladder tissue, inducing pathological changes such as biliary sludge deposition, transient ischemia, and cholestasis, which ultimately results in cholecystitis (da Fonseca et al., 2014). Pazopanib and axitinib are oral TKIs. Pazopanib targets VEGFR-1, -2, and -3, PDGFR-α and -β, and the stem cell factor receptor c-Kit, whereas axitinib predominantly inhibits VEGFR-1, -2, and -3 (Keating, 2015; Frampton, 2017). As with other VEGFR-TKIs, suppression of VEGFR signaling can precipitate microthrombosis and ischemia, which are considered key contributors to the development of acalculous cholecystitis (Elice et al., 2009; Kamba and McDonald, 2007). Notably, biliary epithelial cells secrete VEGF and express high levels of VEGFR-2 and -3; blockade of these receptors by VEGFR-TKIs impairs biliary epithelial proliferation and induces cholestasis, thereby promoting local inflammation and cholecystitis (Sanda et al., 2011; Gaudio et al., 2006). Furthermore, TKIs including pazopanib have been associated with bile acid-dependent hepatotoxicity, which may compromise bile transport and exacerbate bile stasis, predisposing the gallbladder to inflammatory injury (Saran et al., 2022).

## 4 Conclusion

In summary, as commonly used targeted therapies for RCC, pazopanib and axitinib rarely induce cholecystitis as an adverse reaction, making it prone to clinical oversight. This case highlights the need for clinicians to heighten vigilance regarding rare adverse reactions when prescribing such TKIs. Enhanced symptom monitoring and diagnostic evaluations during treatment are essential. Should cholecystitis-related manifestations occur, prompt interventions such as drug discontinuation and symptomatic treatment should be initiated. Concurrently, for patients experiencing such adverse reactions, alternative treatment strategies should be explored to ensure therapeutic safety and improve patient prognosis, thereby providing a reference for individualized targeted therapy in RCC.

## Data Availability

The original contributions presented in the study are included in the article/supplementary material, further inquiries can be directed to the corresponding author.
